# AMPK agonist alleviate renal tubulointerstitial fibrosis via activating mitophagy in high fat and streptozotocin induced diabetic mice

**DOI:** 10.1038/s41419-021-04184-8

**Published:** 2021-10-09

**Authors:** Ya-chun Han, Shi-qi Tang, Yu-ting Liu, Ai-mei Li, Ming Zhan, Ming Yang, Na Song, Wei Zhang, Xue-qin Wu, Can-hui Peng, Hao Zhang, Shikun Yang

**Affiliations:** 1 Department of Nephrology, The Third Xiangya Hospital, Central South University, Changsha, Hunan China; 2 Department of Nephrology, The Second Xiangya Hospital, Institute of Kidney Disease, Central South University, Changsha, Hunan China; 3 International Medicine Department, Ningbo First Hospital, Zhejiang University, Ningbo, China; 4 Department of Laboratory Medicine, The Third Xiangya Hospital, Central South University, Changsha, China

**Keywords:** Diabetes complications, End-stage renal disease, Experimental models of disease

## Abstract

Renal tubulointerstitial fibrosis was a crucial pathological feature of diabetic nephropathy (DN), and renal tubular injury might associate with abnormal mitophagy. In this study, we investigated the effects and molecular mechanisms of AMPK agonist metformin on mitophagy and cellular injury in renal tubular cell under diabetic condition. The high fat diet (HFD) and streptozotocin (STZ)-induced type 2 diabetic mice model and HK-2 cells were used in this study. Metformin was administered in the drinking water (200 mg/kg/d) for 24 weeks. Renal tubulointerstitial lesions, oxidative stress and some indicators of mitophagy (e.g., LC3II, Pink1, and Parkin) were examined both in renal tissue and HK-2 cells. Additionally, compound C (an AMPK inhibitor) and Pink1 siRNA were applied to explore the molecular regulation mechanism of metformin on mitophagy. We found that the expression of p-AMPK, Pink1, Parkin, LC3II, and Atg5 in renal tissue of diabetic mice was decreased obviously. Metformin reduced the levels of serum creatinine, urine protein, and attenuated renal oxidative injury and fibrosis in HFD/STZ induced diabetic mice. In addition, Metformin reversed mitophagy dysfunction and the over-expression of NLRP3. In vitro pretreatment of HK-2 cells with AMPK inhibitor compound C or Pink1 siRNA negated the beneficial effects of metformin. Furthermore, we noted that metformin activated p-AMPK and promoted the translocation of Pink1 from the cytoplasm to mitochondria, then promoted the occurrence of mitophagy in HK-2 cells under HG/HFA ambience. Our results suggested for the first time that AMPK agonist metformin ameliorated renal oxidative stress and tubulointerstitial fibrosis in HFD/STZ-induced diabetic mice via activating mitophagy through a p-AMPK-Pink1-Parkin pathway.

## Introduction

Diabetic nephropathy (DN) is one of the most severe microvascular complications in diabetic patients [1]. Approximately 30–40% of patients with diabetes mellitus (DM) develop nephropathy and progression to renal injury [2]. It significantly decreases the quality of life in people with diabetes. Unfortunately, current therapeutic strategies still cannot effectively inhibit the progression of DN. Besides, the pathogenesis of DN was still not known clearly. Recently, studies have demonstrated that renal tubulointerstitial fibrosis could occur during the early stages of DN [3, 4]. As renal tubule contains abundant mitochondria, normal mitochondrial function was crucial for a good functionality kidney. Much more importantly, emerging pieces of evidence have showed that mitochondrial dysfunction play a critical role in the DN tubulointerstitial damage, and potential novel therapies that ameliorate damaged mitochondria should have beneficial effects on DN [5].

Mitophagy was a special type of autophagy, which could selectivity eliminate disrupted and dysfunctional mitochondria [6]. It was an important way of mitochondrial quality control. If damaged mitochondria had not been removed by mitophagy, the accumulation of mitochondrial fragments would result in excessive generation of mitochondrial ROS and oxidative stress. An accumulating body of evidence has indicated that mitophagy governs the mitochondrial quality control and cell fate [7]. Notably, some previous studies have showed that mitophagy dysfunction was associated with DN, and some agents targeted on mitophagy have been shown beneficial effects [8, 9]. However, both the upstream regulatory signaling and the downstream effector molecule of mitophagy was far from clear. Emerging evidence showed that Adenosine monophosphate activated protein kinase (AMPK) might had a far-reaching regulatory effect on mitochondrial homeostasis in metabolic disorders, more importantly, AMPK was closely related to autophagy, especially mitophagy [10]. Thus it could be seen that AMPK-mitophagy pathway might represent an attractive intervention target for DN. Given these facts, we performed this study to explore the effects and mechanisms of AMPK-mediated mitophagy in diabetes-induced renal tubulointerstitial fibrosis.

## Research design and methods

### Cell lines, antibodies, and reagents

Human proximal tubular epithelial cells (HK-2) was stored in the Institute of Kidney disease, Central South University using liquid nitrogen container. Streptozocin (STZ) was purchased from Sigma-Aldrich (USA). Metformin (HY-17471A) was purchased from MedChem express (USA). Other reagents and antibodies were shown in the Supplementary File (Supplementary Research design and methods).

### Animal experimental design

A total of 24 eight-week-old C57BL/6 mice (~25 g B.W) were purchased from HUNAN SJA Laboratory Animal Co,.LDT (Hunan, China), then they were divided randomly into three groups. The first group was fed with a normal diet sustainably for 24 weeks. The second group and the third group was fed with a high fat diet (HFD). After 4 weeks of HFD feeding, mice of group 2 and group 3 were single injected intraperitoneally with STZ for once (100 mg/kg body weight). Moreover, the third group mice was administered metformin in the drinking water (200 mg/kg) every day for 24 weeks. More details of animal experimental design was shown in the Supplementary Research design and methods. The animal experiments was approved by the Ethics Review Committee of The Third Xiangya Hospital, Central South University.

### Cell culture and treatment

Briefly, HK-2 cells were exposed to media containing different concentrations of D-glucose (5, 30 mM) and with or without other interventions for indicated time (72 h). In addition, for gene disruption, HK-2 cells were pretransfected with PINK1 siRNA using Lipofectamine 3000 (Invitrogen, USA) in accordance with the manufacturer’s protocol.

### Renal morphological analyses and histological staining

Renal tissues (*n* = 3 mice per group) was stained with hematoxylin-eosin (H&E), Periodic acid-Schiff (PAS) and Masson’s staining. Glomerular lesions and interstitial lesions were analyzed using a semiquantitative scoring system in accordance with the established histopathological classification for DN [11].

### Confocal microscopy

A LSM 780 META laser scanning microscope (Zeiss LSM 780) was used to complete the confocal microscopy examination as previously described. The Image J software was used for images analysis.

### Electron microscopy examination

The mitochondrial morphology in renal cortices and cultured HK-2 cells were detected using TEM in accordance with the manufacturer’s protocol [12].

### Mitochondrial isolation

The mitochondria in renal tissue or HK-2 cells was isolated using mitochondrial extraction kit in accordance with the manufacturer’s protocol [13]. More details of the experimental methods and design were shown in the Supplementary File (Supplementary Research design and methods).

### Statistical analysis

All statistical analysis was performed using the SPSS 22.0 software and GraphPad Prism 7.0. *T*-test was performed to compare the differences between two groups. The one-way analysis of variance (ANOVA) with Tukey’s post hoc analysis was used to compare the results between more than two groups. *P* < 0.05 was defined as statistically significant.

## Results

### General parameters and renal pathological changes of diabetic mice with metformin treatment

**Fig. 1:**
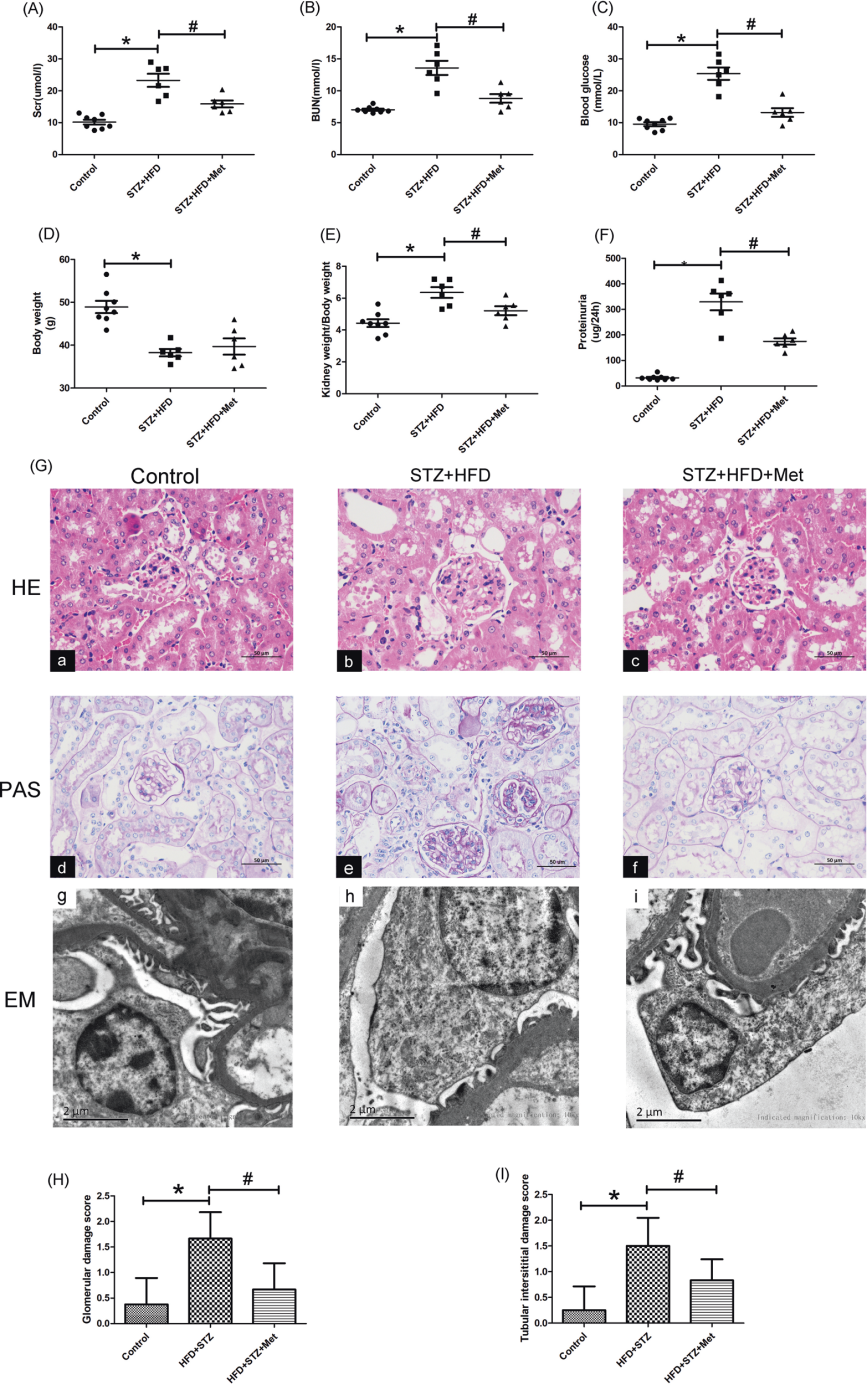
Effects of metformin treatment on biochemical index and renal pathological changes in the kidney of HFD/STZ induced diabetic mice. **A**–**C** Serum Cr, BUN and blood glucose levels of three groups mice at 24 weeks after STZ injection. **D** Body weight of three groups mice at 24 weeks after STZ injection. **E** The kidney weight/body weight of three groups mice at 24 weeks after STZ injection. **F** Twenty-four hours proteinuria content of three groups mice at 24 weeks. Values are presented as the mean ± SD, *P < 0.05 vs. control group, ^#^*P* < 0.05 vs. STZ + HFD group, *n* = 8. **G** Renal tissue sections stained with H&E (**a**–**c**) and PAS (**d**–**f**) (magnification ×400). Electron microscopy (EM) analysis showed that significant ultrastructural changes in diabetic mice including the fusion of the foot processes and the thickening of the glomerular basement membrane. While these injuries were obviously alleviated by metformin treatment. (**g**–**i** magnification ×10,000), *n* = 3. **H** Glomerular damage scores. **I** Tubulo-interstitial damage scores, *P < 0.05 vs. control group, ^#^*P* < 0.05 vs. STZ + HFD group, *n* = 3.

During the 24-weeks experiment, two mices in HFD/STZ group and two mices in HFD/STZ treatment with metformin group died. The biochemical indexes of renal function were observed in this study. We found that the levels of Scr (Fig. 1A) and BUN (Fig. 1B) were increased significantly in HFD/STZ mice. In addition, the levels of blood-glucose, 24 h urinary protein and the ratio of kidney weight/body weight were elevated significantly in all diabetic mice, and metformin treatment could restore these changes (*P* < 0.05, Fig. 1C, E, F). PAS staining showed that renal glomerular basal membrane thickening and mesangial matrix proliferation (Fig. 1G). Furthermore, TEM detection showed that there were significant ultrastructural changes in diabetic mice including the fusion of the foot processes and the thickening of the glomerular basement membrane. While these injuries were obviously alleviated by metformin treatment as indicated by glomerular damage and tubulo-interstitial damage scores (*P* < 0.05, Fig. 1H, I).

### Metformin prevented diabetes-induced oxidative stress and renal interstitial fibrosis

**Fig. 2:**
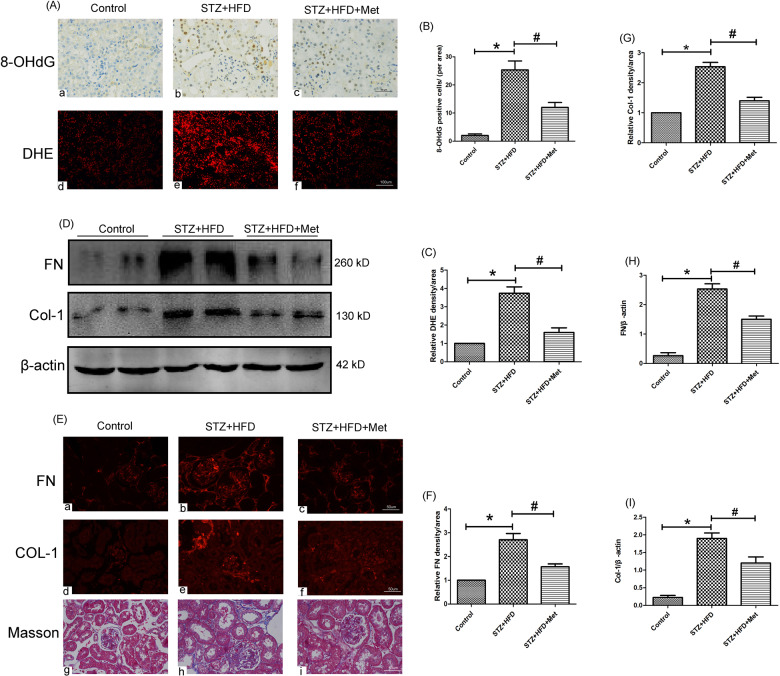
Effects of metformin on renal oxidative stress and renal interstitial fibrosis in HFD/STZ induced diabetic mice. **A** IHC analysis of 8-OHdG (magnification ×400, upper panel) and DHE staining (lower panel) in mouse renal tissue of three groups (magnification ×200). **B**, **C** Bar graphs representing quantification of tissues stained with 8-OHdG (**B**) and DHE (**C**), *P < 0.05 vs. control groups, ^#^*P* < 0.05 vs. STZ + HFD groups, *n* = 3. **D** Western blot analysis of FN (upper panel) and Col-1(middle panel) protein expression in the renal tissue of three groups. **E** IF analysis of FN (upper panel) and Col-1 (middle panel) in mouse renal tissue of three groups (magnification ×400), renal tissue sections are stained with Masson (lower panel, magnification ×400). **F**, **G** Semiquantification of IF staining for FN (**F**) and Col-1 (**G**). **H**–**I** Densitometric analyses of the Western blotting results, FN to β-actin (**H**), Col-1 to β-actin (**I**). Values are presented as the mean ± SD, *P < 0.05 vs. control group, ^#^*P* < 0.05 vs. STZ + HFD group, *n* = 3.

IHC detection showed that renal 8-OHdG level was obviously increased in diabetic mice (Fig. 2A). Moreover, DHE staining was significantly increased in the renal tissue of diabetic mice (Fig. 2A), which was an index of ROS generation. While these changes were significantly reversed by metformin treatment (*P* < 0.05, Fig. 2A–C). Besides, Masson staining showed that obviously increased renal tubulo-interstitial fibrosis in the kidney of diabetic mice (Fig. 2E). Furthermore, IF staining revealed that the expression of FN and Col-1 were notably increased in the diabetic mice (Fig. 2E), while metformin treatment could obviously alleviate these tubulointerstitial lesions (*P* < 0.05, Fig. 2E–G). To confirm the above findings, western blot analysis was carried out, similar result was observed regarding FN and Col-1 protein expression (Fig. 2D, H, I). The protein ladders of western blot for FN, Col-1 and β-actin in Fig. 2D was shown in Supplementary Figure legends (Appended Fig. 1).

### Effect of metformin on the expression of AMPK, p-AMPK, NLRP3, and IL-1 β in kidney of diabetic mice

**Fig. 3:**
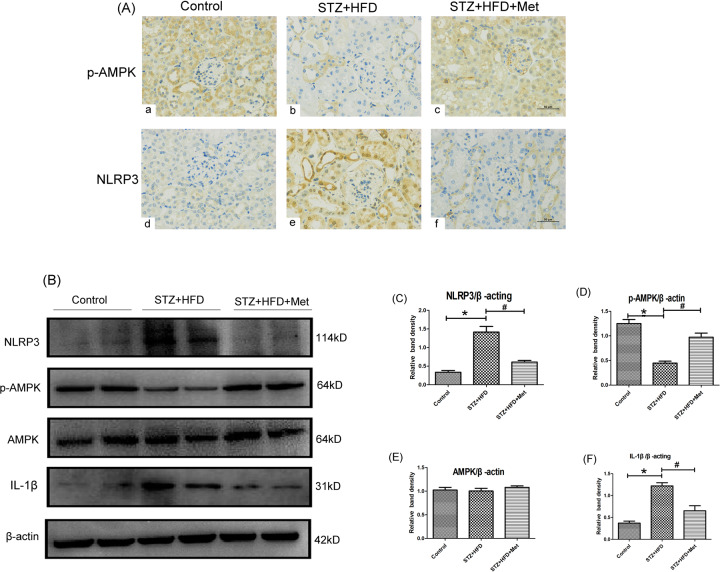
Renal AMPK, p-AMPK, and NLRP3 expression in HFD/STZ induced diabetic mice following metformin treatment. **A** Renal IHC staining with anti-p-AMPK antibody (upper panel) and anti-NLRP3 antibody (lower panel) (magnification ×400). **B** Western blot analysis of NLRP3 (upper panel), p-AMPK, AMPK (middle panel), and IL-1βprotein (lower panel) expression. **C**–**F** Densitometric analyses of the Western blotting results, NLRP3 to β-actin (**C**), p-AMPK to β-actin (**D**), AMPK to β-actin (**E**), IL-1β to β-actin (**F**). Values are presented as the mean ± SD, *P < 0.05 vs. control group, ^#^*P* < 0.05 vs. STZ + HFD group, *n* = 3.

As shown in Fig. 3, IHC staining showed that the protein level of NLRP3 increased significantly in the renal tubular epithelial cell of diabetic mice (Fig. 3A-d, e). Conversely, we found that the expression of p-AMPK was decreased obviously in the renal tissue of diabetic mice (Fig. 3A-a, b). After treatment with metformin for 24 weeks, the level of NLRP3 decreased markedly in the diabetic mice (Fig. 3A-e, f). While metformin treatment increased the expression of p-AMPK in the kidney of diabetic mice. To confirm the above results, we performed western blot analysis using renal tissues of various group, similar result was found regarding NLRP3 and p-AMPK protein expression (Fig. 3B). To further confirm the role of metformin in inflammation under diabetic nephropathy, we detected the expression of IL-1β in renal tissue. Western blot analysis indicated the level of IL-1β increased markedly in the diabetic mice. While metformin treatment decreased the expression of IL-1β (*P* < 0.05, Fig. 3F). Conversely, there was no obvious difference in AMPK expressions in control and HFD/STZ induced diabetic mice and type 2 diabetic mice treated with metformin (Fig. 3B, E). Additionally, quantitative analysis of western blot confirmed the reduction of NLRP3 and upregulation of p-AMPK by metformin therapy (*P* < 0.05, Fig. 3B–D). The protein ladders of western blot for NLRP3, p-AMPK, AMPK, IL-1β, and β-actin in Fig. 3B was shown in Supplementary Figure legends (Appended Fig. 2).

### Metformin reversed HFD/STZ-caused mitophagy dysfunction in kidney of diabetic mice

**Fig. 4:**
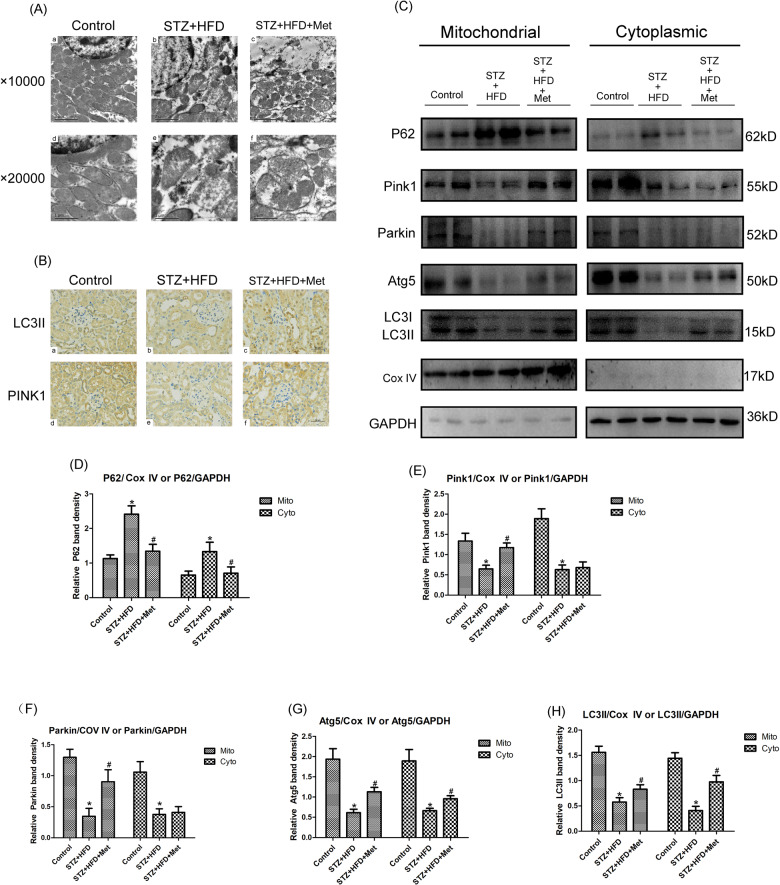
Effects of metformin on mitophagy dysfunction in renal tubular cell of HFD/STZ induced diabetic mice. **A** EM analysis showed obvious mitochondrial morphological changes in diabetic mice renal cells, such as mitochondria swelling and fragmentation, these changes were partially reversed by metformin therapy. In addition, metformin treatment group exhibited conspicuous mitochondrial autophagosome compared with the diabetic mice group (**a**–**c**, magnification ×10,000, upper panel; **d**–**f**, magnification ×20,000, bottom panel). **B** Renal IHC staining with anti-LC3II antibody (upper panel) and anti-Pink1 antibody (lower panel) (magnification ×400). **C** Western blot analysis of P62 (upper panel), Pink1, Parkin, Atg5 (middle panels), and LC3II (bottom panel) protein expression in mitochondria (left panels) and cytoplasm (right panels). **D**–**H** Densitometric analyses of the western blotting results, P62 to CoxIV or P62 to GAPDH (**D**), Pink1 to CoxIV or Pink1 to GAPDH (**E**), Parkin to CoxIV or Parkin to GAPDH (**F**), Atg5 to CoxIV or Atg5 to GAPDH (**G**), LC3II to CoxIV or LC3II to GAPDH (**H**). Values are presented as the mean ± SD, *P < 0.05 vs. control group, ^#^*P* < 0.05 vs. STZ + HFD group, *n* = 3.

As shown in Fig. 4, TEM detection showed that the tubular mitochondria of diabetic mice exhibited deformations, such as mitochondria swelling and fragmentation (Fig. 4A-b, e). These changes were significantly alleviated by metformin treatment. In addition, the renal tubular cell in diabetic mice treated with metformin exhibited conspicuous mitochondrial autophagosome compared with the diabetic mice group (Fig. 4A-c, f). Furthermore, we evaluated the expression of key proteins related to mitophagy. As shown in Fig. 4B, IHC staining indicated that the protein levels of LC3II and Pink1 decreased obviously in the renal tubular epithelial cell of diabetic mice (Fig. 4B-b, e). After treatment with metformin for 24 weeks, the levels of LC3II and Pink1 increased markedly (Fig. 4B-c, f). To evaluate whether mitophagy functioned properly, some other mitophagy-related factors in mitochondria and cytoplasm has been detected respectively. Western blot analysis showed that HFD/STZ treatment markedly downregulated the protein expressions of Pink1 and Parkin both in mitochondria and cytoplasm, metformin treatment provoked an increase in mitochondrial Pink1 and Parkin protein levels (*P* < 0.05, Fig. 4C, E, F), while metformin had no remarkable effect on cytoplasmic Pink1 and Parkin protein levels (*P* < 0.05, Fig. 4C, E, F), which indicated that these factors were translocated from cytoplasm to mitochondria when AMPK agonist metformin stimulation happened. Besides, we have found that the levels of LC3-II and Atg5 both in mitochondria and cytoplasm were dramatically increased by metformin treatment (*P* < 0.05, Fig. 4C, G, H), while the expression of P62 was decreased in the metformin treatment group (*P* < 0.05, Fig. 4C, D), suggesting that the mitophagy was inhibited in diabetic condition, while AMPK agonist metformin therapy could promote the occurrence of mitophagy in the renal tubular cell.The protein ladders of western blot for P62, Pink1, Parkin, Atg5, LC3I/II, CoxIV, and GAPDH in Fig. 4C was shown in Supplementary Figure legends (Appended Fig. 3).

### Metformin restored mitophagy in HK-2 cells exposed to HG/HFA conditions

**Fig. 5:**
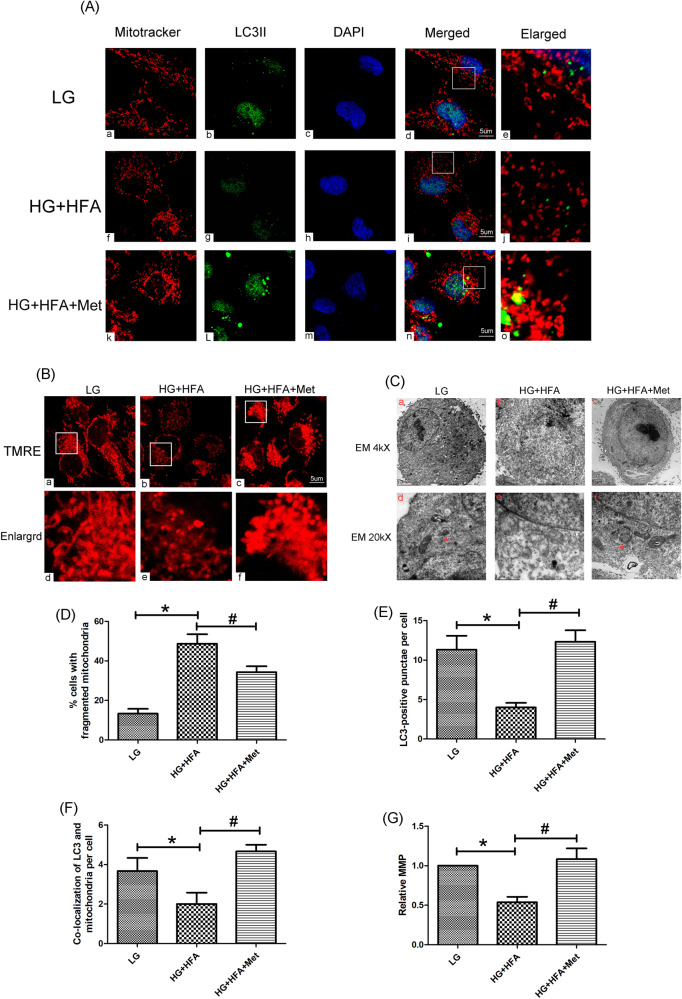
Effects of metformin on mitochondrial morphology, mitophagy and MMP in HK-2 cells exposed to HG/HFA conditions. **A** Laser-scanning confocal microscopy detection showed that LC3II expression and mitochondrial morphology in HK-2 cells exposed to HG/HFA conditions and pretreated with metformin (magnification ×630). **B** TMRE staining for mitochondrial membrane potential (MMP) in HK-2 cells subjected to HG/HFA treatment with metformin (magnification ×630). **C** TEM analysis showed obvious mitochondrial morphological changes in HK-2 cells under HG/HFA condition, such as mitochondria swelling, these changes were partially reversed by metformin therapy. In addition, metformin treatment group exhibited conspicuous mitochondrial autophagosome compared with the HG/HFA group (**a**–**c** magnification ×4000, upper panel; **d**–**f** magnification ×20,000, bottom panel). **D** Semi-quantification of mitochondrial fragmentation of various group. **E** Semi-quantification of LC3-positive punctate per cell. **F** Semi-quantification for the co-localization of LC3II and mitochondria. **G** Quantification of MMP as measured with TMRE staining. Values are presented as the mean ± SD, *P < 0.05 vs. LG group, ^#^*P* < 0.05 vs. HG/HFA group, *n* = 3.

IF staining indicated that HG/HFA stimulation decreased LC3II expression, and mitotracker staining showed that increased mitochondrial fragmentation in HK-2 cells exposed to HG/HFA conditions (Fig. 5A, D, E). These changes were reversed by metformin treatment. Furthermore, the colocalization of mitochondria (labeled with red) and LC3II (labeled with green) was observed. Treatment of HK-2 cells with metformin for 72 h promoted the colocalization of mitochondria and LC3II (*P* < 0.05, Fig. 5A, F). In addition, TEM detection showed that HK-2 cells treated with metformin exhibited obvious mitochondrial autophagosome (Fig. 5C). It suggested that mitophagy was inhibited in HK-2 cells under HG and HFA conditions, while metformin could promote the occurrence of mitophagy in HK-2 cells. On the other side, in vitro studies showed that exposure to HG and HFA conditions obviously reduced the level of MMP, as indicated by TMRE staining (Fig. 5B), these changes were reversed in HK-2 treated with metformin (*P* < 0.05, Fig. 5B, G).

### Metformin downregulated fibrosis related factors and mitochondrial ROS generation depending on mitophagy in HK-2 cells exposed to HG/HFA conditions

**Fig. 6:**
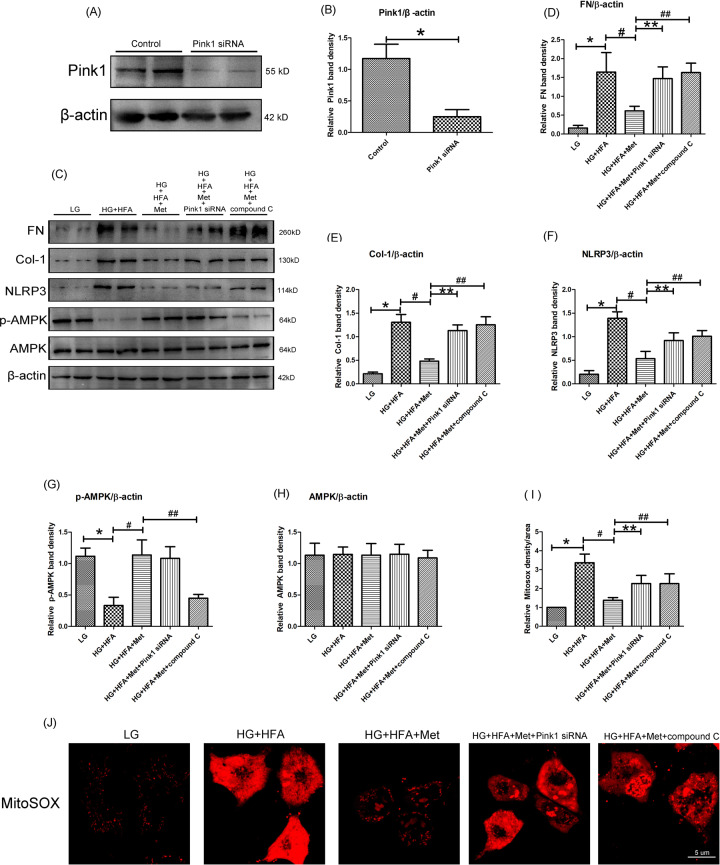
Metformin treatment regulated FN, Col-1, NLRP3, p-AMPK expression, and mitochondrial ROS generation in HK-2 cells exposed to HG/HFA conditions. **A** Western blot analysis of Pink1 protein expression in the HK-2 cells of control and Pink1 siRNA group. **B** Densitometric analyses of WB results, bar graph represents the ratio of Pink1 to β-actin, *P < 0.05 vs. control groups, *n* = 3. **C** Western blot analysis of FN (upper panel), Col-1, NLRP3, p-AMPK (middle panel), and AMPK (bottom panel) protein expression in the HK-2 cells of various groups. **D**–**H** Each bar graph represents the densitometric analyses of FN to β-actin (**D**), Col-1 to β-actin (**E**), NLRP3 to β-actin (**F**), p-AMPK to β-actin (**G**), AMPK to β-actin (**H**). **J** MitoSOX Red staining represented of mitochondrial ROS levels in HK-2 cells of various groups (magnification ×630). **I** Quantification of mitochondrial ROS production as measured with MitoSox Red staining. Values are presented as the mean ± SD, *P < 0.05 vs. LG group, ^#^*P* < 0.05 vs. HG + HFA group, **P < 0.05 vs. HG + HFA + Met group, ^##^*P* < 0.05 vs. HG + HFA + Met group, *n* = 3.

To further explore whether metformin alleviate renal interstitial fibrosis and mitochondrial ROS generation was p-AMPK/Pink1-dependent, compound C (an AMPK inhibitor) and Pink1 siRNA were applied. As expected, the expression of Pink1 in HK-2 cells was significantly reduced by Pink1 siRNA treatment (*P* < 0.05, Fig. 6A, B). Besides, Western blot analysis showed that the protein levels of FN, Col-1 and NLRP3 were increased obviously in the HK-2 cells under HG/HFA condition, and MitoSOX Red staining showed that exposure to HG/HFA condition increased remarkably mitochondrial ROS level (Fig. 6J), while these changes were dramatically reversed by metformin treatment (*P* < 0.05, Fig. 6C–F, I). Conversely, the anti-fibrotic and anti-mitochondrial ROS effects of metformin were abolished by pretreatment with Pink1 siRNA or compound C (*P* < 0.05, Fig. 6C–F, I). Moreover, the metformin induced upregulation of p-AMPK was altered by compound C (*P* < 0.05, Fig. 6C, G), while Pink1 siRNA had no effect on the level of p-AMPK (*P* > 0.05, Fig. 6C, G), it indicated that p-AMPK was an upstream signal molecule of Pink1. The protein ladders of western blot for FN, Col-1, NLRP3, p-AMPK, AMPK, and β-actin in Fig. 6C was shown in Supplementary Figure legends (Appended Fig. 4).

### Metformin restored mitophagy in HK-2 cells exposed to HG/HFA conditions through p-AMPK-Pink1-Parkin pathway

**Fig. 7:**
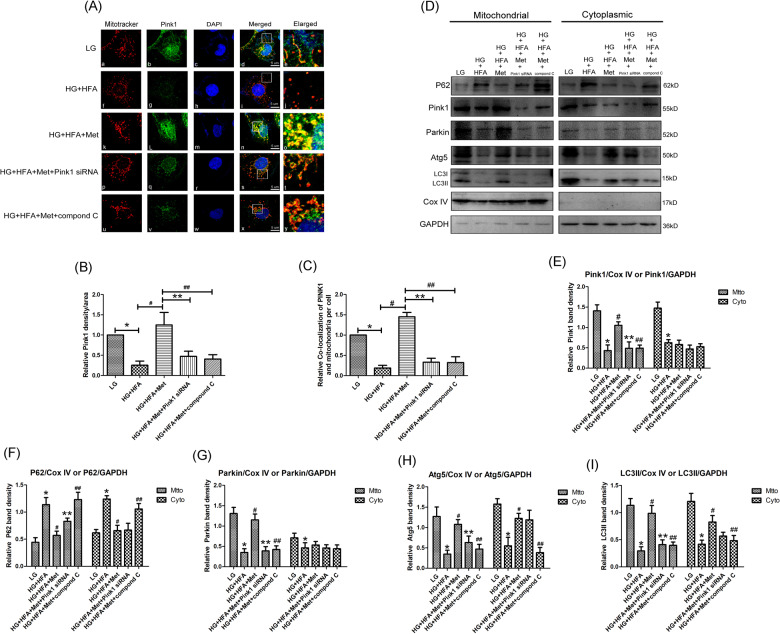
Metformin activated mitophagy in HK-2 cells under HG/HFA conditions through p-AMPK-Pink1-Parkin pathway. **A** Laser-scanning confocal microscopy detection showed Pink1 expression and mitochondrial morphology in HK-2 cells exposed to HG/HFA conditions and pretreated with metformin, Pink1 siRNA or compond C (magnification ×630). **B** Quantification of IF staining for Pink1. **C** Semi-quantification for the co-localization of Pink1 and mitochondria. **D** Western blot analysis of P62 (upper panel), Pink1, Parkin, Atg5 (middle panels), and LC3II (bottom panel) protein expression in mitochondria (left panels) and cytoplasm (right panels) of HK-2 cells. **E**–**I** Densitometric analyses of the western blotting results, Pink1 to CoxIV or Pink1 to GAPDH (**E**), P62 to CoxIV or P62 to GAPDH (**F**), Parkin to CoxIV or Parkin to GAPDH (**G**), Atg5 to CoxIV or Atg5 to GAPDH (**H**), LC3II to CoxIV or LC3II to GAPDH (**I**). Values are presented as the mean ± SD, *P < 0.05 vs. LG group, ^#^*P* < 0.05 vs. HG + HFA group, **P < 0.05 vs. HG + HFA + Met group, ^##^*P* < 0.05 vs. HG + HFA + Met group, *n* = 3.

To explore whether the reduced activity of p-AMPK was associated with diabetes-induced mitophagy dysfunction. IF staining showed that HG/HFA stimulation decreased Pink1 expression (Fig. 7A-f, g). Furthermore, the mitophagy autophagosomes was observed (yellow spotting area) as colocalization of mitochondria (labeled with red) and Pink1 (labeled with green). Treatment of HK-2 cells with metformin for 72 h promoted the colocalization of mitochondria and Pink1 (Fig. 7A, C), which suggested that metformin therapy promoted the translocation of Pink1 from the cytoplasm to mitochondria. We then analyzed whether the translocation of Pink1 to mitochondria could be blocked by AMPK inhibition, as shown in Fig. 7A, the colocalization of mitotracker and Pink1 was inhibited by the pretreatment of Compound C (Fig. 7A-u–y). Similarly, the enhanced effect of metformin on mitochondrial Pink1 deposition was also obviously blocked by Pink1 siRNA. To confirm the above findings, western blot analysis was performed using mitochondria and cytoplasm protein, respectively. We found that the protein expressions of Pink1, Parkin, Atg5, and LC3II was markedly down-regulated both in mitochondria and cytoplasm protein of HK-2 cells exposed to HG/HFA condition, while the level of P62 was increased obviously (*P* < 0.05, Fig. 7D–I), and metformin treatment provoked an increase in mitochondrial Pink1 and Parkin protein levels (*P* < 0.05, Fig. 7D, E, G), while metformin had no remarkable effect on cytoplasmic Pink1 and Parkin protein levels (*P* < 0.05, Fig. 7D, E, G), which further confirmed that AMPK agonist metformin promoting the translocation of Pink1 to mitochondria, and then activating mitophagy in HK-2 cells. To further demonstrate whether metformin regulate mitophagy through an AMPK dependent pathway, AMPK inhibitor Compound C and Pink1 siRNA were used in this study. The metformin induced the upregulation of Pink1, Parkin, Atg5, and LC3II in mitochondrial protein were abolished by pretreatment with Pink1 siRNA or Compound C (*P* < 0.05, Fig. 7D, E, G, H, I). Similar alterations of Atg5 and LC3II protein levels were also observed in cytoplasmic protein from HK-2 cells (*P* < 0.05, Fig. 7D, H, I). Conversely, pretreatment with Pink1 siRNA had no obvious effect on cytoplasmic protein expression of P62, Parkin, and Atg5 (*P* > 0.05, Fig. 7D, F, G, H), these results indicating that p-AMPK was an upstream signal molecule of Pink1, metformin regulated mitophagy through p-AMPK/ Pink1/ Parkin pathway in HK-2 cells. The protein ladders of western blot for P62, Pink1, Parkin, Atg5, LC3I/II, CoxIV, and GAPDH in Fig. 7D was shown in Supplementary Figure legends (Appended Fig. 5).

## Discussion

With the development of DN research, the role of renal tubulointerstitial lesions and interstitial fibrosis in the progression of DN has been widely concerned [14–16]. It has been proposed that high glucose, albuminuria, and advanced glycosylation end products has intrinsic renal tubular toxicity. These are potentially capable of triggering an interstitial inflammatory and productive fibrosis reaction. Consequently contributing to renal interstitial fibrosis [17]. In this study, we have observed obvious tubulointerstitial lesion and fibrosis in the diabetic mice. The mechanism of renal tubulointerstitial injury was very complex and poorly understood, recent research and our preliminary study have showed that mitochondria dysfunctional (e.g., abnormal mitophagy and mitochondrial oxidative stress) play a vital role in this process [4, 5, 18]. It indicating that regulation of mitophagy by exogenous therapeutic agents might alleviate renal tubulointerstitial injury of DN.

Mitochondrial dysfunction was considered as one of the main factors of high glucose induced renal tubular epithelial cell injury in DN. In stressful environment such as hyperglycemia, mitochondria is highly susceptible to damage [19]. In our previous study, we found that the mitochondrial morphology was changed in HK-2 cells induced by high glucose [4]. Mitophagy is a type of macroautophagy specifically targeted toward damaged mitochondria. It is an essential intracellular process that eliminates dysfunctional mitochondria and maintains cellular homeostasis, as damaged mitochondria can release proapoptotic factors and induce the production of ROS. Therefore, mitophagy is postulated to be cytoprotective for DN. However, the role of autophagy in renal tubule injury of DN is still in controversial. Some studies have showed that autophagy has a renoprotective effect for DN, impaired podocyte autophagy would exacerbate proteinuria in DN [20]. Conversely, Zhao et al. reported that liraglutide exerted a renoprotective effect by inhibiting autophagy in the kidney of diabetic rats [21]. As a special and selective autophagy, the impact of mitophagy on the progression of DN has not been fully elucidated. Now there is more and more research have found that tubular mitophagy was strongly linked to the tubulointerstitial injury in DN [22]. Similarly, our data suggested that the activity of renal tubular cell mitophagy was obviously downregulated, while upregulation of mitophagy by metformin might be a key part in alleviating renal interstitial fibrosis in DN.

It is widely appreciated that the level of mitochondria ROS rising dramatically in states of hyperglycemia, and excessive ROS has deleterious effects on renal tissue structure and function [23, 24]. Mitophagy and mitochondria ROS is constantly affecting each other. It has been reported that mitophagy could control mitochondrial ROS through degradation of damaged mitochondria [25]. Conversely, ROS was reported could promote the formation of mitophagy [26]. Our study showed that in the renal tubular cell of DN mice, activating mitophagy by metformin therapy could obviously suppress mitochondrial ROS and subsequent reduce NLRP3 inflammasome activation. Our previous research proposed that activation of the NLRP3 inflammasome played an important role in the pathogenesis of AKI [27]. Furthermore, the impact of NLRP3 inflammasome on DN had also gained a growing number of concerns [28]. In line with this, our study showed the protein levels of NLRP3 and IL-1β increased markedly in the diabetic mice. While metformin treatment decreased the expression of NLRP3 and IL-1β. Previous studies generally suggested that mitochondrial ROS was an activator of the NLRP3 inflammasome [29]. Here, we demonstrated that Pink1-Parkin mediated mitophagy played a pivotal role in the NLRP3 inflammasome activation in DN condition. When exposed to high glucose ambience, a deficiency of Pink1 decreased the level of mitophagy and promoted mitochondrial ROS production, ultimately leaded to NLRP3 inflammasome activation. While this upregulated expression was blunted by metformin therapy.

AMPK is a highly conserved regulator of energy balance in metabolic stress (e.g., diabetic condition) [30]. Previous studies have shown that the reduced activity of AMPK was associated with mitochondria dysfunction, whereas various pharmacological activator of AMPK (e.g., metformin) gave remarkable nephroprotective effects in diabetic models [31]. Metformin is an old drug widely used for type 2 diabetes patients [32, 33], it is very effective in lowering blood glucose in patients with type 2 diabetes with minimal side-effects. Metformin is also being recommended in the treatment of obesity and metabolic syndrome [34, 35]. In addition, the beneficial effect of metformin on renal interstitial fibrosis has caused more and more attention [36, 37]. In our study, we found that metformin treatment could alleviate renal tubulointerstitial injury in HFD/STZ induced diabetic mice. Likewise, we observed that after hyperglycemia induction via STZ injection, the mice exhibited increased Scr and proteinuria, and enhanced expression of oxidative stress and fibrosis markers. Interestingly, all of these changes were reversed by metformin treatment. However, we found two mice in both the diabetic group and the metformin-treated group died during the 24-weeks experiment, which was probably induced by the overlong experiment time.

It has been found that metformin therapy could reduce hepatic gluconeogenesis and lower the blood glucose. Likewise, we found that high glucose level was relieved by metformin treatment in diabetic mice. Recent studies have confirmed that metformin elicited its therapeutic effects mainly via activation of AMPK pathway. It directly activated intrarenal AMPK and subsequently ameliorated relevant intracellular pathways associated with renal epithelial cells and endothelial dysfunction [38]. To confirm the protective effect of metformin was mainly depending on AMPK activation, we performed in vitro studies using HK-2 cells. Increased mitochondrial ROS and fibrosis index has been observed in HK-2 cells exposed to HG/HFA condition. In addition, AMPK agonist has been shown to attenuate these changes. Additional studies were performed using compound C, a cell-permeable AMPK inhibitor, this agent significantly reversed the effects of metformin. Collectively, the improvement of renal injury observed in STZ/HFD induced mice treated with metformin could be explained by both the improvement of metabolic derangements and the amelioration of local oxidative stress via activating AMPK.

The AMPK regulation of autophagy in diabetic complications has been extensively studied over the past years [30]. As an AMPK agonist, metformin has been found to rescue mitophagy in human renal proximal tubular epithelial cells under high glucose environment [39]. Moreover, recently, Wang et al. found metformin could improve mitochondrial respiratory activity through activation of AMPK [40]. On the contrary, the effect of metformin on AMPK expression and mitophagy level was poorly investigated in the renal tubular cell. Perhaps the most intriguing result from our research was decreased mitochondrial translocation and expression of Pink1 following AMPK inhibition. This finding prompted the speculation that AMPK phosphorylation might occur as an upstream target of Pink1. A recent study performed by Wang et al. found that AMPKα2 could specifically activate the phosphorylation of Pink1 at Ser495 in cardiocytes, subsequently, phosphorylated Pink1 recruited the E3 ubiquitin ligase, Parkin, to depolarized mitochondria, and then enhanced the role of the Pink1-Parkin pathway involved in cardiac mitophagy [41]. In our study, we found AMPK agonist promoted the transferring of Pink1 from cytoplasm to mitochondria, although we did not evaluate the phosphorylation level of Pink1, we have the enough reason to believe that AMPK-Pink1-Parkin pathway was a pivotal regulatory pathway of mitophagy in DN.

There was some limitations to the present study. First, the metformin regulation of autophagy and mitophagy in diabetic complications has been studied previously. This study is to further determine the effect of metformin on AMPK expression and mitophagy level on the basis of previous research. Second, mitochondrial quality control was a very complex process containing mitochondrial ROS, mitophagy, and mitochondrial fragmentation, in this study, the effects of metformin on other mitochondrial function (such as regulating mitochondrial dynamics or biogeneration) has not been investigated. Third, in our study, metformin was administered in the drinking water instead of intragastric administration, this might resulting in inaccurate dose administration of metformin.

## Conclusion

In conclusion, the present study revealed that AMPK might act as a vital regulator in the process of diabetic renal tubulointerstitial injury. It indicating that AMPK agonist metformin could alleviate renal tubulointerstitial fibrosis via activating mitophagy through a AMPK-Pink1-Parkin pathway and reduce mitochondrial damage and ROS generation in diabetic environment.

## Supplementary information

Supplementary File

Appended Figure 1. The protein ladders of western blot for FN,Col-1 and β-actin in Figure 2D.

Appended Figure 2. The protein ladders of western blot for NLRP3, p-AMPK, AMPK, IL-1β and β-actin in Figure 3B.

Appended Figure 3. The protein ladders of western blot for P62, Pink1, Parkin, Atg5, LC3I/II, CoxIV and GAPDH in Figure 4C.

Appended Figure 4. The protein ladders of western blot for FN, Col-1,NLRP3, p-AMPK, AMPK and β-actin in Figure 6C.

Appended Figure 5. The protein ladders of western blot for P62, Pink1, Parkin, Atg5, LC3I/II, CoxIV and GAPDH in Figure 7D.

aj-checklist

## Data Availability

The data of this study are available from the first author and corresponding author upon reasonable request.
