# Supplementary material for: AMPK agonist alleviate renal tubulointerstitial fibrosis via activating mitophagy in high fat and streptozotocin induced diabetic mice
Source: Cell Death Dis. 2021 Oct 9;12(10):925. doi: 10.1038/s41419-021-04184-8 (PMC8502176; doi:10.1038/s41419-021-04184-8)
Supplement: Supplementary file 1 — Supplementary File [file 41419_2021_4184_MOESM1_ESM.doc]

**Supplementary File**

**Supplementary Research design and methods**

**Antibodies and reagents:** Human proximal tubular epithelial cells (HK-2) was stored in the Institute of Kidney disease, Central South University using liquid nitrogen container. Streptozocin (STZ) was purchased from Sigma-Aldrich (USA). Metformin (HY-17471A) was purchased from MedChem express (USA). Anti-AMPKa1+AMPKa2 (ab131512), anti Collagen-1 (Col-1, ab34710 and ab270993) and anti-β-actin antibody (ab8226) were obtained from Abcam (UK). The anti phospho-AMPKa (#2535), rabbit IgG antibody to Pink1(#6946) and mouse IgG antibody to Parkin (#4211) were obtained from Cell Signaling Technology (USA). Anti-8-OHdG antibody (GTX41980) was purchased from Gene Tex InC (USA). Rabbit IgG antibody to LC3II (ab48394) was obtained from Abcam (UK). The anti-SQSTM1/P62 (18420-1-AP), anti-GAPDH (60004-1-Ig), anti-COXIV (11242-1-AP) and anti-Atg5 (10181-2-AP) were from Proteintech, while the anti-SQSTM1/P62 (#8025) reacted with mice was obtained from Cell Signaling Technology. Rabbit polyclonal IgG antibody to NLRP3 (ab214185) was obtained from Abcam (UK). The rabbit IgG antibody directed against Fibronectin (FN, AF-5335) antibody was obtained from Affinity Biosciences (USA). Secondary antibodies were purchased from KangChen Bio-tech (Shanghai, China). Other materials, including DMEM/F12 medium, bovine serum albumin (BSA) were purchased from GIBCO (USA). Mitosox Red mitochondrial superoxide indicator (M36008) and Dihydroethidium (DHE, D11347) were obtained from Thermo Fisher Scientific (USA). High fat rodent diet that contains 60 kcal% fat was purchased from Research Diets, Inc (New Brunswick, Canada).

**Animal experimental design:** A total of 24 eight-week-old C57BL/6 mice (~25 g B.W) were purchased from HUNAN SJA Laboratory Animal Co,.LDT (Hunan, China), then they were divided randomly into 3 groups. The first group was fed with a normal diet sustainably for 24 weeks. The second group and the third group was fed with a high fat diet (HFD). After 4 weeks of HFD feeding, mice of group 2 and group 3 were single injected intraperitoneally with STZ for once (100 mg/kg body weight). Then the blood glucose levels were tested after 1 week of STZ injection, the blood glucose level of more than 16.7mmol/l was considered as the standard for diabetic model. Low does of STZ would be re-injected intraperitoneally until the blood glucose level of all mice were more than 16.7mmol/l. Moreover, the third group mice was administered metformin in the drinking water (200 mg/kg) every day for 24 weeks. At the end of 24 weeks following the onset of STZ-induced diabetes, all mice were killed to harvest the sera and kidney samples.

**Renal morphological analyses and histological staining:** Renal tissues (n=3 mice per group) were perfused with 4% formaldehyde solution, after deparaffinization and rehydration, then the 4um slice of renal tissue was stained with hematoxylin-eosin (H&E), Periodic acid-Schiff (PAS) and Masson’s staining. Glomerular lesions and interstitial lesions were analyzed using a semiquantitative scoring system in accordance with the established histopathological classification for DN. Specifically, a score of 0 was given if interstitial fibrosis and tubular atrophy (IFTA) was not present in the cortex. A score of 1 was given if less than 25% IFTA was present. A score of 2 was given when at least 25% but less than 50% IFTA was present. Finally, a score of 3 was assigned when at least 50% IFTA was present. In a similar manner, glomerular injury was semiquantitative scoring from 0 to 4 based on mesangial expansion and glomerulosclerosis.

**Serum biochemical index analysis**: A blood glucose monitor (ROCHE ACCU-CHEK, Germany) was used to test the blood glucose every two weeks. 24 hours urine of all mice was collected in individual metabolic cages, and then we used a mouse albumin ELISA kit (Bethyl Laboratories, USA) to test urine albumin concentrations. In addition, the levels of serum creatinine (Scr) and blood urea nitrogen (BUN) were measured using an automated biochemical analyzer (Hitachi 7600, Japan).

**Immunohistochemistry (IHC) and immunofluorescence (IF) detection of the kidney tissue:** Mouse renal tissue sections (n=3 mice per group, 3 µm thick) was prepared, after de-paraffinized, rehydrated, antigen retrieval and blocked the binding of nonspecific antibodies, the renal slice was incubated with various first antibodies: 8-OHdg (1:100 dilution), p-AMPK (1:100 dilution), NLRP3 (1:100 dilution), PINK1(1:100 dilution), LC3II (1:100 dilution), after incubation overnight, secondary antibodies conjugated with peroxidaseand was added. Finally, the diaminobenzidine (DAB) color working solution was added, and the section stained with brown color was considered the sign of a positive expression. Furthermore, we performed IF analysis of FN (1:100) and Col I (1:100) by using 4% formaldehyde-fixed, paraffin-embedded renal tissue sections (3um thick). We used quantitative statistics to count the number of 8-OHdG staining positive cells in three different groups. While the optical densities of various index was calculated using Image J software, and then the fluorescence density value in control group was set as the reference group, finally we calculated the relative density of FN and Col-1 for three time.

**RNAi:** Small interfering RNA (siRNA) transfection was performed with 20 nM siRNA using Lipofectamine 3000 according to the manufacturer's instructions (Guangzhou RIBOBIO CO., LTD). Cells were harvested 72 h after transfection. The siRNAs targeted the following sequences: Pink1A (5’-GGACGCTGTTCCTCGTTAT-3’), Pink1B (5’-CCAACAGGCTCACAGAGAA-3’), and Pink1C (5’-GGCTGGTGATCGCAGATTT-3’). The sequences of negative control (referred to as siRNA control) was not been reported.

**Western blotting Studies**: Firstly, the protein was extracted from renal tissue or HK-2 cell or isolated mitochondria, the protein concentration was quantified using the BCA method. Equal amounts of protein was transferred onto a nitrocellulose membrane, and then analyzed by SDS-PAGE as previous described. Briefly, after incubated with various primary antibodies: anti-FN (1:1,000), anti-Col1 (1:1,000), anti-Pink1 (1:1,000), anti-Parkin (1:1,000), anti-phospho-AMPKa (1:1000), anti-AMPKa1+AMPKa2 (1:1000), anti-P62 (1:1000), anti-LC3II (1:500), anti-Atg5 (1:1000), anti-NLRP3 (1:1000), anti-GAPDH(1:1000), anti-COXIV(1:1000) and anti-β-actin (1:1,000), then the membrane was immersed in solutions containing secondary antibodies. Finally, we used the ECL system (Amersham, USA) for autoradiograms, the band densities of various proteins was compared with β-actin or GAPDH using Image J software.

**Cell culture and treatment:** The HK-2 cells were cultured in DMEM/F12 medium with 10% foetal bovine serum at 37℃ as previously described. Briefly, HK-2 cells were exposed to media containing different concentrations of D-glucose (5, 30 mM) and with or without other interventions: metformin(500uM), compound C (20uM) for indicated time (72h). In addition, for gene disruption, HK-2 cells were pretransfected with PINK1 siRNA using Lipofectamine 3000 (Invitrogen, USA) in accordance with the manufacturer’s protocol.

**Cells IF examination**: After the intervention of various reagent. HK-2 cells were first immersed in MitoTracker Red (1:1000) solution, then the cells were fixed with 4% PFA for 5 minutes, and permeabilized with ice-cold methanol for 10 minutes at -20℃, blocked with blocking buffer for 1 h at 22℃. After these process, the cells were incubated with primary antibody against LC3II (1:100 dilution) or Pink1(1:100 dilution) solution for 2 h at 22℃. The cells were then incubated with FITC- conjugated secondary antibody solution, followed by the counterstaining with DAPI, cells were examined used a confocal laser scanning microscope (Zeiss LSM 780), and then the fluorescence density value in control group was set as the reference group, finally we calculated the relative fluorescence density of LC3II and Pink1 for three times.

**Confocal microscopy**: A LSM 780 META laser scanning microscope (Zeiss) was used to complete the confocal microscopy examination as previously described . The Image J software was used for images analysis.

**Electron microscopy examination**: The mitochondrial morphology was detected using electron microscopy. Briefly, 1mm^3^ pieces of fresh renal cortices was prepared, then renal cortices were fixed with electron microscopy fixative, and then 60-80 nm ultrathin sections was cutted. Lastly thin sections was subjected to uranium lead double staining. Transmission Electron Microscopy (TEM) was used to delineate the mitochondrial morphology in renal tubular cells. Additionally, the mitochondrial morphology in cultured HK-2 cells was detected using TEM in accordance with the manufacturer’s protocol.

**Detection of** **mitochondrial membrane voltage potential (MMP, ΔΨm) and mitochondrial ROS:** HK-2 cells from various experimental groups was stained with TMRE or MitoSOX and then examined by confocal microscopy to evaluate the perturbations of MMP (ΔΨm) or mitochondrial ROS levels respectively. In addition, frozen section of renal tissue was stained with DHE to evaluate the level of intracellular ROS, the Image J software was used for images analysis, and then the fluorescence density value in control group was set as the reference group, finally we calculated the relative fluorescence density of TMRE, MitoSOX and DHE for three times.

**Mitochondrial isolation**: The mitochondria in renal tissue or HK-2 cells was isolated using mitochondrial extraction kit. Briefly, the renal tissue or HK-2 cells were put in ice cold isolation buffer, then the renal tissue or HK-2 cells were homogenized in isolation buffer using a glass homogenizer. The sample was then centrifuged twice for 5 minutes (1000×g, 4℃). Supernatant was transferred to new tubes and centrifuged at 12 000g for 10 minutes (4℃), then after resuspended, pooled, and re-centrifuged, the mitochondrial precipitation was resuspended using 100ul store buffer. Isolated mitochondria were then stored on ice before the start of further research. All steps in this experiment were performed on ice or at 4℃.

**Statistical analysis**: All statistical analysis was performed using the SPSS 22.0 software and GraphPad Prism 7.0. The experimental data was expressed as the mean ± SD, t-test was performed to compare the differences between two groups. The one-way analysis of variance (ANOVA) with Tukey’s post hoc analysis was used to compare the results between more than two groups. P<0.05 was defined as statistically significant.

**Supplementary Figure legends**

**Appended Figure 1.** The protein ladders of western blot for FN,Col-1 and β-actin in Figure 2D.

**Appended Figure 2.** The protein ladders of western blot for NLRP3, p-AMPK, AMPK, IL-1β and β-actin in Figure 3B.

**Appended Figure 3**. The protein ladders of western blot for P62, Pink1, Parkin, Atg5, LC3I/II, CoxIV and GAPDH in Figure 4C.

**Appended Figure 4**. The protein ladders of western blot for FN, Col-1, NLRP3, p-AMPK, AMPK and β-actin in Figure 6C.

**Appended Figure 5.** The protein ladders of western blot for P62, Pink1, Parkin, Atg5, LC3I/II, CoxIV and GAPDH in Figure 7D.
