# Supplementary figures and images for: AMPK agonist alleviate renal tubulointerstitial fibrosis via activating mitophagy in high fat and streptozotocin induced diabetic mice
Source: Cell Death Dis. 2021 Oct 9;12(10):925. doi: 10.1038/s41419-021-04184-8 (PMC8502176; doi:10.1038/s41419-021-04184-8)

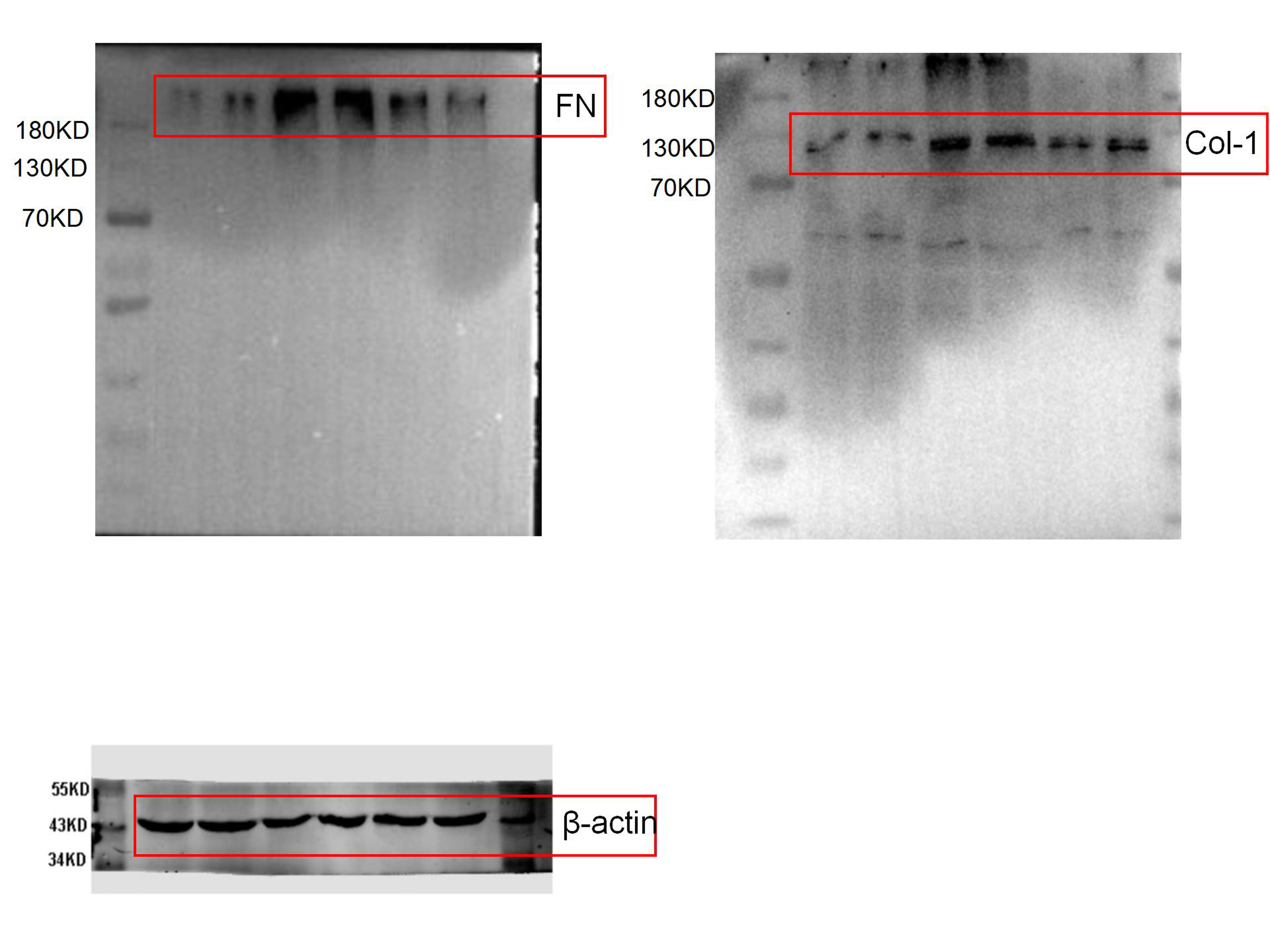

Supplement: Supplementary file 2 — Appended Figure 1. The protein ladders of western blot for FN,Col-1 and β-actin in Figure 2D. [file 41419_2021_4184_MOESM2_ESM.jpg]

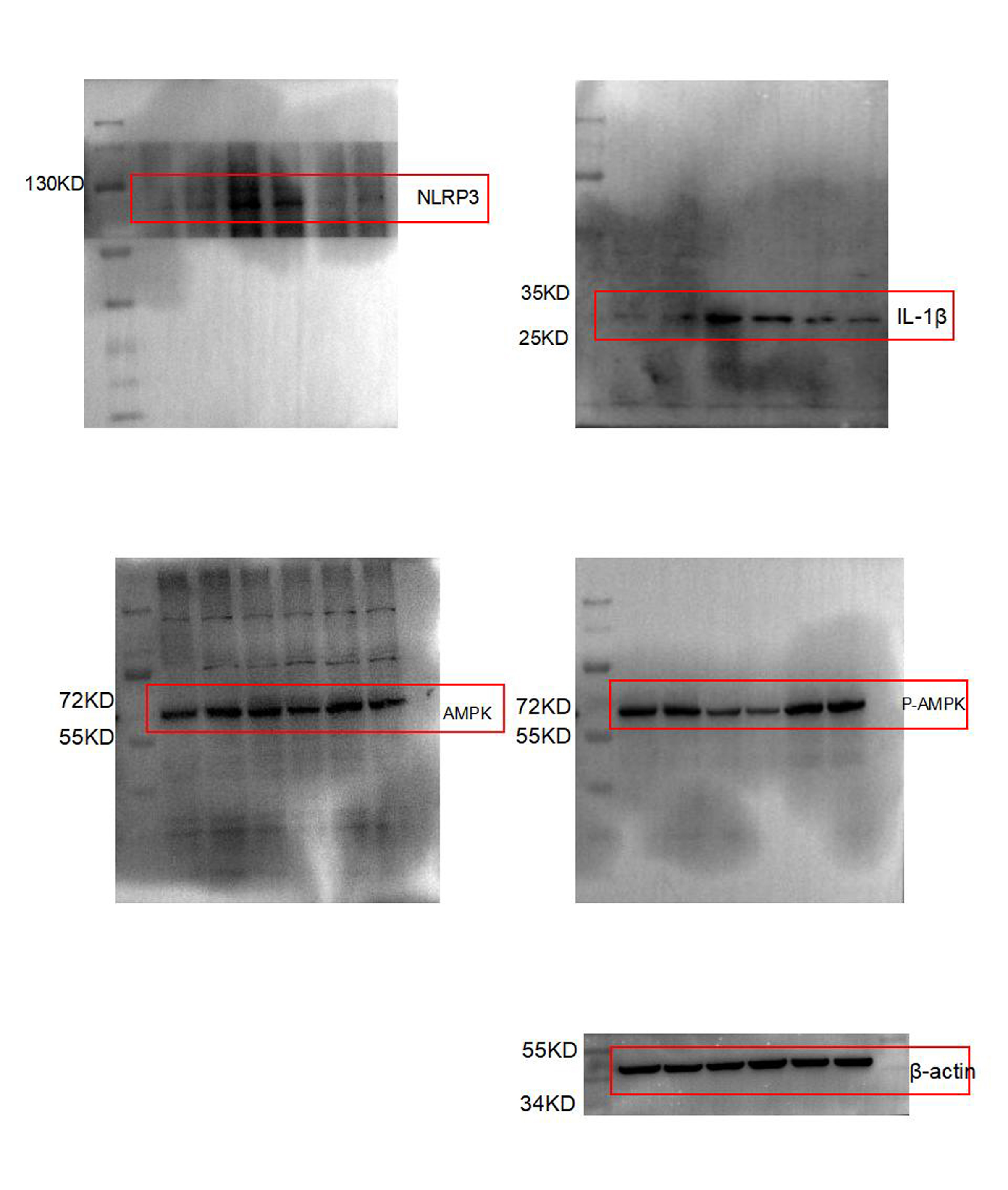

Supplement: Supplementary file 3 — Appended Figure 2. The protein ladders of western blot for NLRP3, p-AMPK, AMPK, IL-1β and β-actin in Figure 3B. [file 41419_2021_4184_MOESM3_ESM.jpg]
